# Influence of muscle mass and bone mass on the mobility of elderly women: an observational study

**DOI:** 10.1186/1471-2318-14-13

**Published:** 2014-01-31

**Authors:** Gláucia R Falsarella, Ibsen B Coimbra, Caroline C Barcelos, Isabele Iartelli, Kedma T Montedori, Manuela NJ Santos, Anita L Neri, Arlete MV Coimbra

**Affiliations:** 1 Gerontology Program, State University of Campinas (UNICAMP), Barão Geraldo, Campinas-SP, Brazil; 2 Department of Medical Clinics, State University of Campinas (UNICAMP), Barão Geraldo, Brazil; 3 Family Health Program, State University of Campinas (UNICAMP), Rua Vital Brasil, 50, Barão Geraldo 13083-888, Campinas-SP, Brazil

**Keywords:** Elderly, Muscle mass, Bone mass, Mobility

## Abstract

**Background:**

The purpose of this study was to investigate the influence of muscle mass and bone mineral density on markers of mobility in dwelling elderly women.

**Methods:**

This cross-sectional study included 99 elderly women, who were 65 years old or above, in Campinas-SP, Brazil. To collect data, we used sociodemographic data, the body mass index (BMI), health status, comorbidities, use of medications, mobility tests (TUG and gait speed) and examinations of the body composition (densitometry with dual-emission X-ray absorptiometry “DXA”). In order to examine the relationship between muscle and bone mass with mobility (gait speed and TUG), we applied the Spearman correlation coefficient.

Also was applied the analysis of covariance (ANCOVA) adjusted for age and comorbidities. To identify the factors associated with mobility, we used the univariate and multivariate logistic regression analysis. The level of significance for statistical tests was P < 0.05.

**Results:**

The correlation between sarcopenia and bone mineral density with mobility tests showed a significant relationship only between sarcopenia and TUG (r = 0.277, P = 0.006) in Spearman correlation coefficient. The result of the correlation analysis (ANCOVA) showed that sarcopenia was associated with gait speed (r^2^ = 0.0636, P = 0.0018) and TUG (r^2^ = 0.0898, P = 0.0027). The results of the multivariate analysis showed that age (P = 0.034, OR = 1.081) was associated with worse performance on gait speed. By highlighting the TUG test, the results of the multivariate analysis showed that the age (P = 0.004, OR = 1.111) and BMI in overweight (P = 0.011, OR = 7.83) and obese (P < 0.001, OR = 7.84) women were associated with lower performance of the functionality of the lower limbs.

**Conclusion:**

The findings with regard to mobility tests which were analyzed in this study indicate the association of variables related to the aging process that contribute to the decline in physical performance, for example, age, BMI and sarcopenia.

## Background

The changes in body composition associated with aging represent potential conditions that favor functional limitations [1]. Musculoskeletal alterations trigger limitations in mobility [2], which also extend to restrictions on the participation in daily activities, difficulties in implementing self-care tasks and absenteeism [3], resulting in higher dependence [4] with negative effects on the quality of life of older adults [5,6].

Loss of muscle mass and low bone mineral density are considered as indications of functional decline [7]. The association between sarcopenia and osteoporosis may be due to the combination of various etiologic factors, such as mechanical aspects; denervation; mitochondrial dysfunction; vitamin D deficiency; low levels of testosterone, estrogens, sulfate of dihydroepiandrostenedione (S-DHEA) and insulin growth factor I (IGF-I); inflammation (elevated IL-6 and TNF-α); and decreased food intake [8,9].

Modifications of the morphophysiological muscle tissue favor decreases in mass, strength and muscle function, which impacts mobility, increases the risk of falls and contributes to fragility [9]. There are variations in the loss of muscle mass according to the age, gender and ethnicity; however, this process starts at approximately 45 years old and, above 80 years old, sarcopenia is present in more than 50% of the elderly population [10].

Another important aspect of the body composition related to advanced age refers to the imbalance between bone formation and resorption that may result in decreased bone mass. Considered as a disease that is associated with bone remodeling, osteoporosis results in greater fragility of the bone tissue and increases the risk of falls and fractures in the elderly population [11,12].

Loss of mobility is one of the major consequences of the deterioration of the musculoskeletal system. Elderly people with restrictions on their mobility have higher rates of falls, chronic disease, dependency, institutionalization and death [13]. Within this framework, we highlight the importance of assessing sarcopenia through measures of physical performance, with the purpose of preventing or delaying the onset of frailty, disability and mortality among the elderly population [7,9,14].

Changes in the body composition related to aging are related mainly to decreases in muscle mass and bone mass. In this sense, the purpose of this study was to investigate the influence of muscle mass and bone mass on the functionality and, specifically, on markers of mobility in elderly patients.

## Methods

### Subjects

This cross-sectional study included 99 elderly women ≥ 65 years old, who were living in Campinas-SP, Brazil. In order to obtain a non-institutionalized sample with a heterogeneous body composition, we chose to include elderly women who were randomly recruited in a clinical environment and in the community from three different sources: 1) community-dwelling elderly participants in a survey about frailty in elderly Brazilians (Study FIBRA Campinas); 2) elderly individuals recruited from the Reference Center on Aging Health (CRI); and 3) ambulatory elderly individuals from the Rheumatology Department of a public hospital in the city.

We excluded elderly individuals who had severe cognitive impairment that would hinder the examinations, patients with functional limitations that prevented ambulation and patients with inflammatory rheumatic diseases. The exclusion was based on the evaluation of the rheumatologist responsible for the assessment, the statement of the elderly people or their caregiver and/or the observation of the researcher. This study was approved by the Ethics Committee of UNICAMP with protocol number 913/2009.

### Sociodemographic and anthropometric data

The investigation included the following variables: age, education, weight, height and body mass index (BMI, calculated according to height/weight^2^, following the categorizations of the World Health Organization) [15]: underweight (<18.5), normal (18.5-24.9), overweight (25.0-29.9), and obese (> 30.0).

### Health conditions

Patients self-reported the presence of the following chronic conditions: cardiovascular disease, stroke, diabetes, hypertension, arthritis or rheumatism, lung disease, cataracts, depression and thyroidopathy. Health conditions were classified into two categories, i.e., ≤4 or >4 diseases. The reporting as to the use of medications assessed the use of ≤4 or >4 medications per day.

### Gait speed

This test represented a geriatric assessment instrument and indicated the functions of daily life activities. The Gait speed showed the time taken to travel a distance of 4.6 meters. Patients would walk a total distance of 4.6 meters, with the initial 2 m and final 2 m disregarded when calculating the time spent in motion (acceleration and deceleration time). Three measurements were performed, and their average value was calculated [16,17].

### Functional testing of mobility and dynamic balance “Timed Up and Go” (TUG)

This test is an effective method to evaluate the mobility and quantify the locomotor performance [18], and it is a predictor of the risk of falls and of the dynamic equilibrium of the elderly people [19]. The individual is requested to rise from a chair (45 cm) and walk as quickly and safely as possible for 3 m in a straight line and then walk back to the chair and sit as in the initial position. Literature categorizes this test on three levels from the run time: ≤10 seconds (TUG 1); between 10.01 and 20 s (TUG 2); and over 20.01 s (TUG 3) [14]; however, this research considered the time of the test as a continuous variable.

### Densitometry with dual-emission X-ray absorptiometry (DXA)

All subjects were evaluated with the Body Dual X-ray Absorptiometry (DXA) software version 4.7 (GE/Lunar Radiation), model DPX-IQ. These analyses were performed by an operator who was blinded to the study objectives. This imaging technique evaluates body composition (muscle, fat and bone mass) [14]. For our study, we analyzed muscle mass and bone mineral density, which allowed the diagnoses of sarcopenia, osteopenia and osteoporosis.

The participants were categorized into two groups by checking their muscle mass: normal or with sarcopenia. In order to calculate the sarcopenia, we applied the following formula: appendicular muscle mass/height^2^ (aLM/h^2^). The cutoff used in this research was 5.45 kg/m^2^[8,10].

In relation to bone mineral density, we calculated the total bone mass as the individual value obtained by Total-BMD1 (g/cm^2^) and Total-Adult2 (T-score). Following the standardized diagnostic criteria suggested by WHO, the participants were separated into three groups with respect to the number of standard deviations (SD) of the T-score: normal (more than −1 SD), osteopenia (−1 SD to −2.5 SD) and osteoporosis (less than - 2.5 SD) [20].

In order to perform this examination, the elderly patients were requested to interrupt the use of medications with calcium 48 hours before the test.

### Statistical analysis

We determined the descriptive statistics. In order to examine the relationship between muscle mass and bone mineral density with mobility tests (gait speed and TUG), we applied the Spearman correlation coefficient and ANCOVA (adjusted for aged and comorbidity). For the univariate and multivariate regression analysis, stepwise criterion for variable selection, we identified the factors associated with mobility. In this analysis, we considered the following variables: age, sarcopenia, bone mass, BMI, comorbidities and medication. The level of significance for statistical tests was 5% or P < 0.05. Statistical analysis was used with the SAS System for Windows (Statistical Analysis System), version 8.02.

## Results

The descriptive analysis of the sample is presented in Table 1. This research included a sample of 99 elderly women with ages ≥ 65 years old (mean of 73.03 years old ± 6.51). The average number of years of schooling was 3.86 years (± 3.57); 66.67% of the sample was not married, and 66.67% reported their race as Caucasian. When considering the health conditions of women, 71.72% described themselves as having four or less diseases, and 28.28% had more than four diseases. Regarding the use of drugs administered, 45.45% used up to four medications a day, while 54.55% used more than four medications a day.

**Table 1:** Characterization of the sample regarding age, schooling, weight, height, bmi, comorbidities, gait speed, TUG, sarcopenia and BMD in elderly women*

	**Mean**	SD**	Min**	Q1**	**Median**	Q3**	Max**
**Age (years)**	77.03	6.51	66.00	72.00	77.00	81.00	94.00
**Schooling (years)**	3.86	3.57	0.00	1.00	4.00	4.00	15.00
**Height (m**^**2**^**)**	1.51	0.05	1.40	1.47	1.51	1.53	1.67
**Weight (kg)**	66.19	16.78	37.00	53.10	64.50	75.50	132.00
**BMI (kg/m**^**2**^**)**	29.12	7.02	16.01	23.37	28.77	32.68	57.89
**Diseases**	3.66	1.67	0.00	2.00	4.00	5.00	8.00
**Gait speed (m/s)**	6.06	2.94	3.26	4.18	5.16	7.17	19.72
**TUG (s)**	13.89	5.58	6.87	9.98	12.43	16.16	36.04
**Sarcopenia kg/m**^**2**^	7.44	1.33	5.36	6.32	7.33	8.11	11.29
**Total BMD (g/cm**^**2**^**)**	1030	120.4	762.0	940.0	1037	1123	1283

*n = 99.

**sd = standard deviation, min = minimum, q1 = quartile 1, q3 = quartile 3, max = maximum.

The body composition analysis indicated an average BMI of 29.12 kg/m^2^ (±7.02). When investigating muscle mass, we found that 68.69% of the participants not presented with sarcopenia, and the average value of muscle mass found in the sample was 7.44 kg/m^2^ (±1.33). In relation to total bone mass, 42.42% did not show any changes in their bones, 36.36% had osteopenia and 21.21% had been diagnosed with osteoporosis.

The descriptive statistics reported regarding the execution time of the mobility tests showed that the average gait speed was 6.06 (±2.94), while for the TUG test, the average speed of the studied participants was 13.89 (±5.58).

The correlation between the variables of sarcopenia and bone mineral density with mobility tests (gait speed and TUG) showed a significant relationship only between sarcopenia and TUG (r = 0.277, P = 0.006), i.e., the lower the muscle mass, the greater the value observed in the TUG test performance (Table 2). The analysis of covariance in Table 3 adjusted for aged and comorbidity, showed a significant association between sarcopenia and gait speed (P = 0.0102) and TUG (P = 0.0027). In Table 4 the analysis of covariance multiple for gait speed and TUG, controlling for age and comorbidities indicated association between sarcopenia and gait speed (r^2^ = 0.0636, P = 0.0018) and TUG (r^2^ = 0.0898, P = 0.0027).

**Table 2:** Correlation between muscle mass and bone mass with gait speed and TUG

	**Sarcopenia**	**Total BMD (g/cm**^**2**^**)**	**Total adult2Tscore**
Gait speed*	r = 0.18281	0.06163	0.08019
n = 98	P = 0.0716	0.5466	0.432
**TUG**	0.27749	0.15157	0.14129
n = 96	0.0062	0.1404	0.1697

*r = Spearman correlation coefficient; P = P value; n = number of subjects.

**Table 3:** Analysis covariance between muscle mass and bone mass with gait speed and TUG

**Gait speed**		**TUG**	
**Sarcopenia**	P*	**Sarcopenia**	P*
Continuous variable	0.0102	Continuous variable	0.0027
**Total BMD (g/cm**^**2**^**)**	P*	**Total BMD (g/cm**^**2**^**)**	P*
Continuous variable	0.2910	Continuous variable	0.0644

*Value-p refers to ANCOVA, adjusted for aged and comorbidity.

**Table 4:** Analysis of covariance multiple for gait speed and TUG, controlling for age and comorbidities

**Gait speed**			
**Sarcopenia**	**Beta (EP)**	P*	**R**^**2 **^**Parcial**
Continuous variable	11.84 (3.68)	0.0018	0.0636
**TUG**			
**Sarcopenia**	**Beta (EP)**	P*	**R**^**2 **^**Parcial**
Continuous variable	6.538 (2.121)	0.0027	0.0898

*Value-p refers to ANCOVA, adjusted for aged and comorbidity.

The univariate logistic regression analysis, which was used to study the relationship of factors associated with poorer performance on mobility tests, found that gait speed was statistically significantly correlated only with advanced age. Using a univariate analysis, it was also found that the TUG test showed significant correlation with the variables of BMI and sarcopenia (Table 5).

**Table 5:** Univariate logistic regression analysis for gait speed and TUG

	**Gait speed**			**TUG**		
**Variables**	OR*	CI 95% OR*	**P**	OR**	CI 95% OR**	**P**
**Age**						
Continuous variable	1.081	1.006-1.161	0.034	1.052	0.988-1.120	0.113
**Sarcopenia**						
No (ref.)	1.00	-	-	1.00	-	-
Yes	0.61	0.22-1.71	0.345	0.31	0.13-0.75	0.010
**Total bone mass**						
Normal (ref.)	1.00	-	-	1.00	-	-
Osteopenia	0.39	0.12-1.24	0.111	0.42	0.17-1.06	0.066
Osteoporosis	1.49	0.46-4.51	0.483	1.09	0.36-3.29	0.886
**BMI**						
Normal (ref.)	1.00	-	-	1.00	-	-
Underweight	1.24	0.27-5.68	0.786	0.55	0.17-1.76	0.310
Overweight	1.97	0.36-0.82	0.436	7.29	1.51-35.08	0.013
Obese	2.72	0.78-9.49	0.116	5.43	1.75-16.85	0.003
**Comorbidities**						
0-4 diseases (ref.)	1.00	-	-	1.00	-	-
>4 diseases	0.78	0.27-2.23	0.646	1.21	0.49-2.98	0.685
**Medication number**						
0-4 (ref.)	1.00	-	-	1.00	-	-
>4	1.38	0.55-3.48	0.492	1.33	0.60-2.98	0.486

*OR (odds ratio) = ratio of risk for poor performance (n = 73 elderly women with good performance and n = 25 elderly women with worse performance); CI 95%, OR = 95% interval of confidence for the risk ratio; ref: reference level; n = 98.

**OR (odds ratio) = ratio of risk for poor performance (n = 24 elderly women with TUG 1 (≤10 s), n = 59 elderly women with TUG 2 (10.01 - 20 s) and n = 13 with TUG 3 (over 20.01 s)); CI 95% OR = 95% interval of confidence for the risk ratio; ref: reference level Proportional hazards model. n = 96.

The results of the multivariate analysis with stepwise criterion for variable selection for gait speed found that age was associated with a worse performance on this test. We found that elderly women of an increased age had a higher risk for lower performance in gait speed, with the risk of poor performance increasing by 8.1% for each year of age, (Table 6).

**Table 6:** Multivariate logistic regression analysis for gait speed and TUG

	**Gait speed**			**TUG**		
**Variables**	**P**	OR*	CI 95% OR*	**P**	OR**	CI 95% OR**
**Age (years)**						
Continuous variable	0.034	1.081	1.006-1.161	0.004	1.111	1.034 -1.194
**BMI**						
Normal (ref.)				-	1.00	-
Underweight				0.158	0.41	0.12-1.42
Overweight				0.011	7.83	1.60-38.27
Obese				<0.001	7.84	2.31-24.21

*OR (odds ratio) = ratio of risk for poor performance (n = 73 with good performance and n = 25 with worse performance); CI 95% OR = 95% interval of confidence for the risk ratio. Stepwise criterion for variable selection; n = 98.

**OR (odds ratio) = ratio of risk for poor performance (n = 24 with TUG 1 (≤10 s), n = 59 with TUG 2 (10.01 – 20 s) and n = 13 with TUG 3 (over 20.01 s)). Proportional hazards model. CI 95% OR = 95% interval of confidence for the risk ratio. Stepwise criterion for variable selection; n = 96.

By highlighting the TUG test, the result of the multivariate analysis showed that age and BMI were associated with lower performance of the functionality of the lower limbs. The elderly women who had a worse performance on the test (and thus, were at higher risk) had an increased age (each year of age increased the risk by 11.1%) and BMI (overweight patients had 7.8 times greater risk of mobility limitation, and obese patients had 7.5 times greater risk) (Table 6).

## Discussion

The aging process is characterized by a gradual decline in the physical function [9]; however, it is not entirely clear whether the reduction in the physical function is the exclusive result of changes in body composition [1]. Epidemiological studies have shown inconsistent results when examining the influence of muscle mass and bone mineral density on physical performance in older adults [1,21,22].

Therefore, this research examined the influence of muscle mass and bone mass on the functionality of the lower limbs and found a correlation among components of the body composition and mobility in the elderly patients. In the investigated sample, the lower muscle mass was associated with reduced physical performance on the gait speed and TUG test. This finding is extremely important because muscle mass represents a modifiable physiological parameter, which can be altered through interventions.

Considered as a metabolic and functional component, the body composition undergoes important changes with age, which are expressed mainly by negative variation in fat-free mass and positive variation in fat mass [23], with repercussion on the physical performance in the old age [1]. Thus, a decreased mobility is strongly associated with changes in the body composition, restrictions in participation and increased risk of hospitalization. Physical limitation is a strong predictor of reduced quality of life in the old age [24,25].

Evidence suggests that changes in the body composition contribute to the onset and progression of the disability in the elderly patients [8]. Shin et al. [1] found that fat-free mass is an independent predictor of functional capacity limitation. Reid et al. [13] added that muscle mass is strongly associated with muscle strength and mobility in individuals with advanced age, that is, less muscle mass in the lower extremities indicates greater risk of a decline in mobility.

A survey conducted by Frisoli et al. [8], which analyzed studies on the cross-sectional area of the muscles of the lower limbs, found that elderly patients in the lowest quartile for this component of body composition were more likely to have mobility limitations. In addition, low muscle mass was also linked with worse balance and an increased risk of falls and disability.

With age, there is a change in the composition of muscle tissue and a consequent change in muscle function. This effect triggers loss of type I muscle fibers and a marked reduction in type II muscle fibers, resulting in decreased muscle strength that affects the performance of everyday tasks, such as getting up from a chair, climbing stairs, and reorganizing the body posture after a loss of balance [9].

However, when applied to multivariate analysis, an association between TUG performance with age and BMI was found; only age represented a risk factor for gait speed. In our study, the influence of age in the mobility was similar to the results from other surveys [19,24]. According to Hayes and Johnson [19], there is a tendency for the execution time for mobility to increase considerably with advancing age. Among the possible causes related to aging that interfere with locomotion are loss of balance, decreased range of motion [26], presence of depressive symptoms and decline in cognitive functions [18,19].

Considering the age, this is a variable associated with sarcopenia, lower bone mineral density and, therefore, it is an indication of osteopenia and osteoporosis [8,14,15]. Therefore, the result found by this research may indicate that the assessment of relative sarcopenia (muscle mass adjusted for appendicular body mass) is clinically and physiologically more relevant when compared with absolute sarcopenia (appendicular muscle mass adjusted for height) as a stronger indicator of mobility performance in elderly women [7].

Our research has shown BMI as a risk factor for lower performance in mobility on the TUG test. Despite controversies with regard to BMI and lean mass and bone mass, as independent variables, and also limiting the use of BMI as an indicator of fat mass, studies indicate an association between BMI and the physical performance in the elderly people [1,22]. A study [22] conducted in Hong Kong in 4,000 elderly communities observed that the group of older people with normal weight (BMI = 18.5-22.9 kg/m2) needed less time to perform the 6 m walking test compared with other BMI groups. According to the authors, this result suggests the influence of fat mass on functional limitation in elderly people.

Bohannon et al. [27] also indicated a significantly negative relationship between physical function and anthropometric parameters, such as BMI, waist circumference and waist-hip ratio. In the Nutrition and Function Study, elder and obese patients were twice as likely to have lower performance when analyzing the function of the lower limbs [28].

The findings regarding mobility tests which were analyzed in this study may indicate the influence of other variables related to the aging process that contribute to the decline in physical performance [8], for example, BMI [1,22], fat mass [22,27], balance and visual disorders, medications and comorbidities [1].

This study had some limitations. It is necessary that other investigations with larger samples clarify whether sarcopenia, osteopenia and osteoporosis in isolation are associated with poor performance on mobility tests or whether the relationship exists only with the interaction of minor muscles and low mineral density bone. It is also important that other studies analyze the impact of fat infiltration within the muscle tissue due to the extent to which this interpretation represents a better predictor of physical performance in older adults. Furthermore, were included elderly with chronic diseases, among them some that can affect body composition, such as thyroid.

## Conclusion

The findings with regard to mobility tests which were analyzed in this study indicate the association of variables related to the aging process that contribute to the decline in physical performance, for example, age, BMI and sarcopenia.

## Competing interests

The authors declare that they have no competing interests.

## Authors’ contributions

GRF, IBC and AMVC contributed to the conception and design of the study, analysis and interpretation of data, revising it critically for important intellectual content and final approval of the version to be submitted. ALN contributed to the design of the study. CCB, IL, KTM and MNJS contributed to the drafting of the article and revising it critically for important intellectual content. All authors read and approve the final manuscript.

## Pre-publication history

The pre-publication history for this paper can be accessed here:

http://www.biomedcentral.com/1471-2318/14/13/prepub
